# INTERNALIZED STIGMA – HOW WE VIEW OUR MENTAL ILLNESS

**DOI:** 10.1192/j.eurpsy.2023.2190

**Published:** 2023-07-19

**Authors:** M. Vieira, B. Fonseca Silva, J. Silva Ribeiro

**Affiliations:** Psychiatry, Vila Nova de Gaia/Espinho Hospital Center, Vila Nova de Gaia, Portugal

## Abstract

**Introduction:**

Stigma in mental health settings is described as a set of negative and unrealistic beliefs about those with mental illness. Authors suggest that stigma is consistently underdefined and overused, leading to resources toward preventing and managing this problem lacking intention and efficiency. Three interacting levels of stigma are defined: social, structural, and internalized or self-stigma. Internalized stigma refers to how people with mental illness see themselves as mentally unwell and, therefore, of lesser value.

**Objectives:**

We aim to discuss the impact of internalized stigma on psychiatric patients and ways of prevention and stigma resistance.

**Methods:**

We performed a non-systematic literature review from the data base *PubMed* using the key words “internalized stigma” and “mental illness”.

**Results:**

Internalized stigma is one of the major factors leading to delayed contact with psychiatric care up to two years in outpatients. In psychiatric patients, higher internalized stigma was associated with weakened social support and integration, hopelessness and lower self-esteem and sense of coherence. Low self-esteem is the most significantly associated factor and mediates lower quality of life and higher treatment avoidance. The risk of self-esteem loss seems higher in patients with more insight, especially if they also have a loss of valued social identity. Although some studies suggest higher levels of internalized stigma in female, single and lower educated patients, adjusted statistical analyses do not validate these sociodemographic variations. It is however more prevalent in those with depression and who had been hospitalized because of their mental illness. The impact of internalized stigma is often compared to the levels of the illness burden itself, leading to higher levels of depression and greater psychiatric symptom severity. Additionally, more self-stigma seems to predict suicidal ideation, particularly in young adults.

**Conclusions:**

The internalization of negative stereotypes undermines empowerment and negatively impacts the evolution and recovery of psychiatric patients. There’s strong evidence that general stigma constitutes a risk factor for poor biopsychosocial health outcomes. Programs addressing multiple stigma components seem to be most effective in improving suicide prevention. However, most self-stigma interventions involve groups, which can create barriers for people who are not comfortable disclosing a mental health condition to others. Anti-stigma programs are most effective when they involve people with lived experience of mental health conditions in all aspects of development. Interventions from a younger age should focus on prevention of general stigma by improving understanding of mental illness and reducing self and outwards discrimination. Working on professionals own stigmatizing behaviors is also key to improve the way we communicate and educate populations on how to internally process mental health problems.

**Disclosure of Interest:**

None Declared

